# Vulvovaginal yeast infections, gestational diabetes and pregnancy outcome

**DOI:** 10.1186/s12884-023-05391-1

**Published:** 2023-01-26

**Authors:** Leeni Blomberg, Katri Backman, Pirkka V. Kirjavainen, Anne M. Karvonen, Maijakaisa Harju, Leea Keski-Nisula

**Affiliations:** 1 Institute of Clinical Medicine, School of Medicine, University of Eastern Finland, Kuopio, Finland; 2 Department of Pediatrics, Kuopio University Hospital, University of Eastern Finland, Kuopio, Finland; 3 Department of Health Security, Finnish Institute for Health and Welfare, Kuopio, Finland; 4 Institute of Public Health and Clinical Nutrition, University of Eastern Finland, Kuopio, Finland; 5 Department of Obstetrics and Gynecology, South Karelia Central Hospital, 53130 Lappeenranta, Finland; 6 Department of Obstetrics and Gynecology, Kuopio University Hospital, PL 100, 70029 Kuopio, Finland

**Keywords:** Yeast infection, Vulvovaginal candidiasis, Gestational diabetes mellitus, Neonates, Pregnancy

## Abstract

**Background:**

The primary aim was to evaluate the association between gestational diabetes and blood glucose levels and vulvovaginal yeast infections in pregnancy. Secondly, we clarified the possible associations between maternal and prenatal factors, and birth outcomes and yeast infections.

**Methods:**

Three thousand nine hundred sixty-five pregnant women of the Kuopio Birth Cohort Study (KuBiCo) reported vulvovaginal yeast infections during pregnancy, via electronic questionnaires. Maternal and prenatal data, as well as clinical obstetric and early neonatal outcomes were registered during and after birth. The oral glucose tolerance test was performed on 3,079 women during pregnancy. Logistic regression analysis evaluated the possible multivariable associations between yeast infections, gestational diabetes and other prenatal and maternal factors.

**Results:**

No association was detected between gestational diabetes or blood glucose levels and vulvovaginal yeast infections during pregnancy. In multivariable analysis, women with yeast infections were more often multiparous, with higher education and had used more often antibiotics during pregnancy compared to others. No significant associations were detected in multivariable analysis between infections, the mode of delivery, preterm birth, birth weight or Apgar scores.

**Conclusions:**

Women with reported vulvovaginal yeast infections managed generally well during pregnancy. They had no more gestational diabetes or higher blood glucose levels and their newborns managed equally well during early neonatal period.

## Background

Vulvovaginal yeast infection is highly common in sexually active women. Approximately two out of three women have had some symptoms of infection during their lives [1]. The main predisposing factor to yeast infection is the use of antimicrobial medication that destroys beneficial *Lactobacillus* bacteria of normal vaginal microbiota. Other common predisposing factors are diabetes, immunosuppressive conditions, and genetic predispositions, the use of glucocorticoids, oral contraceptives, or hormone replacement therapy [2–4]. In addition, women suffer more from yeast infections during pregnancy due to high estrogen levels and elevated glycogen levels in vaginal secretions [4, 5]. Some studies have shown that vulvovaginal yeast infection may be associated with the increased risk for preterm birth, and further treatment of asymptomatic infection might reduce the risk of preterm birth [6].

Women with diabetes and those with high blood glucose levels have a higher rate of vulvovaginal yeast infection, according to multiple studies [2, 7, 8]. However, there are only a few studies about the actual connection between gestational diabetes mellitus (GDM) or high blood glucose levels during pregnancy and yeast infection. These studies have shown that women with GDM have more vaginal yeast infections during pregnancy [9, 10]. Kelekci and his group showed that pregnant women with yeast infection had a higher rate of impaired glucose tolerance, measured as higher glucose values via oral glucose tolerance test (OGTT), compared to women without infection; however, they did not find similar results with GDM [11]. An Italian cross-sectional study including 473 pregnant women suggested that diabetes (involving GDM and pregestational diabetes) were associated with the increased the risk for yeast infection during pregnancy in multigravida, but not in primigravida [12]. In contrast, other studies did not show an association between GDM or impaired glucose tolerance during pregnancy and yeast infections [13, 14].

Results of existing studies regarding the association between yeast infection and GDM seem inconsistent, as well as less apparent than previous evidence, considering the increased risk of vaginal candida colonization and infection in diabetic women. The primary aim of this study was to evaluate the association between GDM or high blood glucose levels and vulvovaginal yeast infections during pregnancy, within a large birth cohort study of 3,965 pregnant women. Second, we wanted to clarify whether yeast infections were associated with obstetric and perinatal outcomes.

## Methods

### Study design and setting

This study is a part of the Kuopio Birth Cohort (KuBiCo) research project: its intention is to study the effect of different environmental exposures during pregnancy (for example, medications and nutrition, as well as different lifestyles and environmental factors) on the health and diseases of their offspring later in life [15].

The data in this study was extracted from the KuBiCo database in October 2022 and consisted of coded information gathered from 3,965 pregnant women, giving birth at Kuopio University Hospital (KUH) between January 2014 and June 2022. The overall mean participation rate in KuBiCo during 2014–2022 has been 33.8% of all pregnant women giving birth in KUH.

### Ethical considerations

The study complies with the Helsinki Declaration. KuBiCo research project has been reviewed and approved by The Research Ethics Committee of the Hospital District of Central Finland, Jyväskylä (8.12.2011, K-S SHP Dnro 18U/2011). All women participated voluntarily and gave written informed consent before participation.

### Data source and data collection

The data on vulvovaginal infections and use of antibiotics was collected by electronic questionnaires during the third trimester of pregnancy. Maternal characteristics, pregnancy, birth and early neonatal clinical data were gathered from KUH birth registers.

### Study variables and definitions

#### Assessment of maternal vulvovaginal infection

In the third trimester questionnaires, women were asked about the separate occurrence of vulvovaginal yeast infections during three different trimesters. Occurrence of infection was classified into three groups, based on recurrence: a). No yeast infections during pregnancy, b). One or two yeast infections during pregnancy, and c). Recurrent yeast infections (three or more during pregnancy). This classification was based on the division between occasional and recurrent yeast infection, often considered as equal to or more than three infections in twelve months [16]. Gynecological vaginal examinations with the use of a speculum were done twice during pregnancy; in the first and in the third trimesters in maternity clinics.

#### Birth outcomes

Birth outcomes included duration of gestation at birth (< 37 gestational weeks (preterm) *vs.* ≥ 37 gestational weeks (term)), mode of delivery (vaginal *vs.* Cesarean birth), birthweight (lowest/highest tertile *vs.* others and 10^th^ /90^th^ percentiles vs. others) and Apgar scores at 5 min (low ≤ 7 *vs.* normal scores ≥ 8).

#### Diagnosis of gestational diabetes

In general, GDM was diagnosed according to Finnish national clinical guidelines [17]. The diagnosis is based on the oral glucose tolerance test (OGTT) performed on nearly all pregnant women between the 24^th^ and 28^th^ gestational weeks. Sometimes, the test is performed between the 12^th^ and 16^th^ weeks, or even earlier in women with a high risk of GDM (for example, women with GDM or macrosomic infant in previous pregnancies, glucosuria at the beginning of pregnancy, use of oral corticosteroids, extreme obesity (BMI > 35 kg/m^2^), close relatives with DMII or polycystic ovary syndrome). If the result of OGTT is normal in the first trimester, the test will be redone between the 24^th^ and 28^th^ gestational weeks. According to the Finnish guidelines, OGTT is not essential in nulliparous women who are equal to or younger than 25 years with normal weight (BMI 18.5–25 kg/m^2^) and have not close relatives with DMII; the same applies in primi/multiparas who are equal to or younger than 40 years with a normal BMI and not having GDM or fetal macrosomia in previous pregnancies. Approximately 70% to 80% of pregnant women in Finland have OGTTs during the pregnancy [17]. GDM was recorded from hospital and birth registers by ICD-10 codes O24.4 or O24.9. Pregnant women without OGTT were included as normal women without GDM. Other evaluated variables in univariate analysis were maternal weight gain in pregnancy and blood glucose values (fasting, at 1 and 2 h) in OGTT.

##### Other maternal variables

Maternal characteristics included age, body mass index (BMI; kg/m^2^) in the first trimester, parity and gravidity, nationality, years of education, working status, smoking behavior, and relationship status. Smoking behavior was classified into two groups: no smoking and any smoking during pregnancy. Smokers indulged through the pregnancy or quit smoking during any trimester; non-smokers did not smoke during pregnancy. Possible infertility treatment before an ongoing pregnancy, occurrence of pregestational diabetes or immunological diseases with immunosuppressive medication, and the use of oral or vaginal antibiotics during pregnancy were recorded. Pregestational diabetes was recorded from the hospital and birth register by ICD-10 codes such as O24.0, O24.1, O24.2, O24.3, E10.9, and E11.9. Pregestational immunological diseases were recorded similarly from registers by ICD-10 codes such as G35, G70, E27, K31, K90.0, L40, M05, M06, M08, M32, M35 and M45 (Multiple sclerosis, Myasthenia gravis, Addison´s disease, inflammatory bowel diseases, coeliac diseases, psoriasis, rheumatoid arthritis and ankylosing spondylitis, Sjögren´s disease, systemic lupus erythematosus and other immunological connective tissue diseases).

##### Statistical analysis

Data were analyzed with IBM SPSS Statistics 27, Armonk, NY, USA. The occurrence of infections (Tables 1, 2 and 3) and birth outcomes (Table 4) were evaluated as outcome variables. Explanatory variables for occurrence of infections in univariate analysis (Tables 1 and 2) were maternal age, parity, body mass index (kg/m^2^), number of earlier pregnancies, nationality, education, working status during pregnancy, smoking, possible infertility treatment before ongoing pregnancy, twin pregnancy, pregestational or gestational diabetes, immunological diseases, gestational weight gain, glucose values in OGTT and the use of peroral or vaginal antibiotics. Nominal variables were evaluated with Pearson’s Chi-squared test and continuous variables with analysis of variance. Women with pregestational diabetes were excluded from the data when analyzing the associations between yeast infections and OGTT and GDM results (Table 2). With twins, neonatal data included information for only one newborn.

**Table 1:** Maternal characteristics and vulvovaginal yeast infections during pregnancy among 3,965 women

	**Total N**	**No infections**	**One or two infections**	**Recurrent infections**	***p-value***
Maternal age, years	31.1 (4.9)	31.1 (4.9)	31.0 (5.2)	31.1 (5.2)	0.992
≤ 29	1,558 (39.3)	1,395 (39.3)	127 (39.7)	36 (37.9)	0.973
30–33	1,167 (29.4)	1,043 (29.4)	93 (29.1)	31 (32.6)	
≥ 34	1,240 (31.3)	1,112 (31.3)	100 (31.3)	28 (29.5)	
Maternal BMI before pregnancy, kg/m^2^	25.2 (5.1)	25.2 (5.1)	24.8 (4.8)	26.6 (6.8)	0.016
< 25	2,429 (61.2)	2,183 (61.5)	196 (61.3)	50 (52.6)	0.094
25–30	927 (23.4)	820 (23.1)	84 (26.3)	23 (24.2)	
> 30	609 (15.4)	547 (15.4)	40 (12.5)	22 (23.2)	
Parity	0.8 (1.0)	0.8 (1.0)	0.9 (1.1)	1.0 (1.0)	0.024
Nulli- or primiparous	3,221 (81.2)	2,905 (81.8)	251 (78.4)	65 (68.4)	0.002
Multiparous	744 (18.8)	645 (18.2)	69 (21.6)	30 (31.6)	
Numbers of earlier pregnancies^a^	2.2 (1.5)	2.2 (1.4)	2.4 (1.7)	2.6 (1.5)	0.001
One to two pregnancies	2,697 (68.0)	2,439 (68.7)	206 (64.4)	52 (54.7)	0.005
Equal to three or more pregnancies	1,268 (32.0)	1,111 (31.3)	114 (35.6)	43 (45.3)	
Nationality					
Finnish	3,843 (96.9)	3,438 (96.8)	313 (97.8)	92 (96.8)	0.630
Other	122 (3.1)	112 (3.2)	7 (2.2)	3 (3.2)	
Education, years	16.3 (3.2)	16.3 (3.2)	16.8 (3.4)	16.4 (2.8)	0.025
≤ 14	943 (23.8)	862 (24.3)	62 (19.4)	19 (20.0)	0.211
15–17	1,798 (45.3)	1,603 (45.2)	147 (45.9)	48 (50.5)	
≥ 18	1,224 (30.9)	1,085 (30.6)	111 (34.7)	28 (29.5)	
Working status					
Working full-time	2,718 (68.5)	2,429 (68.4)	220 (68.8)	69 (72.6)	0.882
Working part time	368 (9.3)	334 (9.4)	28 (8.8)	6 (6.3)	
Studying	406 (10.2)	359 (10.1)	37 (11.6)	10 (10.5)	
Not working or studying	473 (11.9)	428 (12.1)	35 (10.9)	10 (10.5)	
Relationship					
Married	2,039 (51.4)	1,824 (51.4)	167 (52.2)	48 (50.5)	0.310
In relationship but not married	1,899 (47.9)	1,704 (48.0)	148 (46.3)	47 (49.5)	
Single	27 (0.7)	22 (0.6)	5 (1.6)	0 (0)	
Smoking during pregnancy	494 (12.5)	437 (12.3)	40 (12.5)	17 (17.9)	0.266
Infertility treatment before ongoing pregnancy	336 (8.5)	304 (8.6)	24 (7.5)	8 (8.4)	0.807
Twin pregnancy	57 (1.4)	54 (1.5)	2 (0.6)	1 (1.1)	0.414
Pregestational diabetes	34 (0.9)	30 (0.8)	4 (1.3)	0 (0.0)	0.495
Immunological diseases ^b^	88 (2.2)	78 (2.2)	10 (3.1)	0 (0)	0.185
Use of peroral antibiotics at any stage of pregnancy	814 (20.5)	653 (18.4)	125 (39.1)	36 (37.9)	< 0.001
Use of vaginal antibiotics at any stage of pregnancy	285 (7.2)	173 (4.9)	91 (28.4)	21 (22.1)	< 0.001
Total	3,965 (100)	3,550 (100)	320 (100)	95 (100)	

Values are N (%) or mean (SD)

^a^Including ongoing pregnancy

^b^Including Addison´s disease (*N* = 2), coeliac disease (*N* = 6), inflammatory bowel diseases (*N* = 36), multiple sclerosis (*N* = 8), myasthenia gravis (*N* = 1), psoriasis (*N* = 2), rheumatoid arthritis and ankylosing spondylitis (*N* = 19), Sjögren´s syndrome, systemic lupus erythematosus and other immunological connective tissue diseases (*N* = 14)

*P*- values are estimated with Pearson's Chi-squared test (between groups) or One-Way ANOVA (continuous variables)

**Table 2:** Association between vulvovaginal yeast infections during pregnancy, gestational weight gain, oral glucose tolerance test results and gestational diabetes mellitus among 3,931 women

	**Total N (%) or mean (SD)**	**No infections**	**One or two infections**	**Recurrent infections**	***p*-value**
Maternal weight gain during pregnancy, kg	13.8 (5.2)	13.8 (5.2)	13.4 (5.0)	14.3 (6.0)	0.378
≤ 10	870 (22.1)	771 (21.9)	82 (25.9)	17 (17.9)	0.086
11–13	854 (21.7)	772 (22.0)	65 (20.6)	17 (17.9)	
14–16	824 (21.0)	742 (21.0)	62 (19.6)	20 (21.1)	
> 16	1,012 (25.7)	912 (25.9)	77 (24.4)	23 (24.2)	
Missing	371 (9.4)	323 (9.2)	30 (9.5)	18 (18.9)	
Oral glucose tolerance test (OGTT) during pregnancy	3,079 (78.3)	2,772 (78.8)	227 (71.8)	80 (84.2)	0.006
Fasting glucose value, mmol/l	4.9 (0.5)	4.9 (0.4)	5.0 (0.8)	4.9 (0.4)	0.478
Missing value in OGTT or OGTT not done	885 (22.5)	786 (22.3)	80 (25.3)	19 (20.0)	
OGTT, 1 h value, mmol/l	7.5 (1.7)	7.5 (1.7)	7.5 (1.7)	7.6 (1.7)	0.895
Missing value in OGTT or OGTT not done	934 (23.8)	827 (23.5)	87 (27.5)	20 (21.1)	
OGTT, 2 h value, mmol/l	6.4 (1.4)	6.4 (1.4)	6.4 (1.3)	6.4 (1.2)	0.898
Missing value in OGTT or OGTT not done	924 (23.5)	820 (23.3)	84 (26.6)	20 (21.1)	
Gestational diabetes ^a^	786 (19.8)	715 (20.3)	50 (15.8)	21 (22.1)	0.141
Total	3,931 (100)	3,520 (100)	316 (100)	95 (100)	

Values are N (%) or mean (SD)

^a^Diagnostic thresholds for gestational diabetes in Finland are 5.3 mmol/l for the fasting stage, 10.0 mmol/l for 1 h or 8.6 mmol/l for 2 h after ingesting the glucose solution

*P*- values are estimated with Pearson's Chi-squared test (between groups) or One-Way ANOVA (continuous variables)

**Table 3:** Adjusted multivariable effects of maternal and prenatal estimates and vulvovaginal yeast infections during pregnancy among 3,965 women

Variable	≥ One infection n/N (%)	Adjusted OR (95% CI)	Recurrent infection n/N (%)	Adjusted OR (95% CI)
Maternal parity				
Nulli- or primiparous	316/3,221 (9.8)	1	65/3,221 (2.0)	1
Multiparous	99/744 (13.3)	1.44 (1.11–1.86)	30/744 (4.0)	1.99 (1.27–3.12)
Maternal BMI before pregnancy, kg/m^2^				
< 25	246/2,429 (10.1)	1	50/2,429 (2.1)	1
25–30	107/927 (11.5)	1.10 (0.85–1.42)	23 /927 (2.5)	1.11 (0.66–1.85)
> 30	62/609 (10.2)	0.92 (0.66–1.28)	22/609 (3.6)	1.72 (0.98–3.00)
Maternal weight gain during pregnancy, kg				
≤ 10	99/878 (11.3)	1	17/878 (1.9)	1
11–13	83/863 (9.6)	0.80 (0.58–1.12)	17/863 (2.0)	1.16 (0.58–2.31)
14–16	83/829 (10.0)	0.84 (0.60–1.16)	20/829 (2.4)	1.45 (0.74–2.85)
> 16	101/1,020 (9.9)	0.84 (0.61–1.15)	23/1,020 (2.3)	1.36 (0.71–2.61)
Missing value	49/375 (13.1)	1.19 (0.81–1.86)	18/375 (4.8)	2.90 (1.45–5.79)
Education, years				
≤ 14	81/943 (8.6)	1	19/943 (2.0)	1
15–17	195/1,798 (10.8)	1.35 (1.01–1.80)	48/1,798 (2.7)	1.42 (0.82–2.45)
≥ 18	139/1,224 (11.4)	1.50 (1.11–2.04)	28/1,224 (2.3)	1.28 (0.70–2.33)
Pregestational diabetes				
No	411/3,931 (10.5)	1	95/3,931 (2.4)	1
Yes	4/34 (11.8)	0.92 (0.30–2.85)	0/34 (0)	–
Gestational diabetes				
No	344/3,179 (10.8)	1	74/3,179 (2.3)	1
Yes	71/786 (9.0)	0.77 (0.58–1.03)	21/786 (2.7)	0.98 (0.58–1.66)
Use of oral antibiotics at any stage of pregnancy				
No	254/3,151 (8.1)	1	59/3,151 (1.9)	1
Yes	161/814 (19.8)	2.69 (2.15–3.38)	36/814 (4.4)	2.11 (1.37–3.25)
Use of vaginal antibiotics at any stage of pregnancy				
No	303/3,680 (8.2)	1	74/3,680 (2.0)	1
Yes	112/285 (39.3)	6.99 (5.31–9.19)	21/285 (7.4)	3.74 (2.24–6.23)

*OR* Odds ratio. All variables entered simultaneously in logistic regression analysis

**Table 4:** Unadjusted and adjusted odds ratios for birth outcomes in 3,965 women in relation to vulvovaginal yeast infections during pregnancy

	N (%) *vs*. N(%)	No infections	One or two infections	Recurrent infections	No infections	One or two infections	Recurrent infections
		(ref.)	OR (95% CI)	OR (95% CI)	(ref.)	aOR (95% CI)	aOR (95% CI)
Cesarean *vs*. vaginal birth	487 (12.3) *vs*. 3,478 (87.7)	1	0.91 (0.63–1.31)	1.57 (0.92–2.67)	1	0.94 (0.65–1.36)	1.71 (0.99–2.94)
Preterm *vs*. term birth	185 (4.7) *vs*. 3,780 (95.3)	1	1.26 (0.76–2.01)	1.42 (0.62–3.29)	1	1.31 (0.79–2.17)	1.64 (0.70–3.84)
Neonatal Apgar scores ≤ 7 *vs.* ≥ 8	137 (3.5) *vs*. 3,828 (96.5)	1	1.11 (0.61–2.04)	1.59 (0.63–3.98)	1	1.17 (0.64–2.15)	1.73 (0.68–4.37)
Birth weight							
1^st^ tertile *vs.* others	1,322 (33.3) *vs*. 2,643 (66.7)	1	0.96 (0.76–1.23)	0.63 (0.39–1.01)	1	0.98 (0.77–1.25)	0.68 (0.42–1.10)
3^rd^ tertile *vs*. others	1,327 (33.5) *vs*. 2,638 (66.5)	1	1.19 (0.94–1.50)	1.29 (0.85–1.97)	1	1.17 (0.91–1.48)	1.15 (0.75–1.76)
≤ 10^th^ percentile *vs.* others	396 (10.0) *vs*. 3,569 (90.0)	1	0.78 (0.52–1.19)	1.04 (0.54–2.03)	1	0.81 (0.53–1.22)	1.14 (0.58–2.23)
≥ 90^th^ percentile *vs.* others	400 (10.1) *vs*. 3,565 (89.9)	1	1.00 (0.68–1.46)	1.43 (0.79–2.59)	1	0.98 (0.66–1.43)	1.26 (0.69–2.31)

OR odds ratio, aOR adjusted odds ratio. *CI* Confidence interval. All outcome variables were adjusted for maternal age, parity, body mass index, maternal pregestational diabetes, infertility treatment and smoking. All variables were entered simultaneously in logistic regression analysis. With twins, neonatal data included information for only one. 1^st^ tertile birth weight ≤ 3,325 g, 3^rd^ tertile birth weight ≥ 3,730 g, ≤ 10^th^ percentile birth weight ≤ 2,892 g, ≥ 90^th^ percentile birth weight ≥ 4,110 g

Logistic regression analysis was used to investigate multivariable simultaneous relationships between outcome variables (any/recurrent yeast infections or birth outcomes) and adjusted effects of various predictors or confounders. Predictors chosen for the logistic regression analysis for yeast infections (Table 3) were gestational diabetes and pregestational diabetes mellitus, and confounding variables which were marginally associated (*p* < 0.10) with the yeast infection in univariate analysis (Tables 1 and 2). Since parity and numbers of earlier pregnancies were highly correlated (Pearson´s correlation *r* = 0.847), only parity was included in the multivariable model. All variables were entered simultaneously into the logistic regression models and were treated as categorical variables. Subanalysis in multivariable logistic regression analysis was done separately in those women who had OGTT performed (*N* = 3,079). The associations between yeast infections and birth outcomes were evaluated by logistic regression analysis with confounders (maternal age, body mass index, parity, pregestational diabetes, infertility treatment before pregnancy and smoking), which were selected before analysis on the basis of earlier reports of their significance in neonatal and birth outcomes [18–20]. All variables were entered simultaneously in analysis and treated as categorial variables (see Table 1). Significance level was set at *p* < 0.05.

## Results

In total, the prevalence of vulvovaginal yeast infection during pregnancy was 10.5% (*n* = 415) among 3,965 women. Of all women, 8.1% (*n* = 320) had one or two infections and 2.4% (*n* = 95) had recurrent infections during pregnancy trimesters. In the first trimester, prevalence of infection was 4.3% (*n* = 170), in the second trimester 6.8% (*n* = 271), and in the third trimester 5.0% (*n* = 197).

Table 1 shows univariate associations between maternal characteristics and infections. Women with recurrent infections had higher mean BMI values compared to other women (26.6 kg/m^2^ *vs.* 25.2 kg/m^2^; *p* < 0.01). Multiparous women and those with a high number of earlier pregnancies had more often (4.0% and 3.4%, consequently) recurrent infections *vs.* nulliparous or primiparous women (2.0%; *p* < 0.001) and women with a lower number of earlier pregnancies (1.9%; *p* < 0.005). In addition, 37.9% to 39.1% of women with yeast infections had used oral antibiotics during pregnancy, compared to 20.4% of women without infections (*p* < 0.001). Similar and even stronger associations were detected with the use of vaginal antibiotics (Table 1).

The OGTT was performed on 78.3% (*n* = 3 079) of the study women and GDM was diagnosed in 19.8% (*n* = 786) (Table 2). OGTT was performed more often on women with recurrent infections *vs.* those with one or two infections, or without infection (84.2% *vs.* 71.8% *vs.* 78.8%; *p* < 0.006). In contrast, women who were not tested were more normal-weighted (mean (SD) BMI 22.6 (3.3) kg/m^2^) and younger (30.1 (5.0) years) compared to women with OGTTs (25.9 (5.3) kg/m^2^; *p* < 0.0001 and 31.4 (4.8) years; *p* < 0.0001). No associations were detected between the prevalence of women with or without infections and GDM (*p* = 0.141) or OGTT results (Table 2). In sensitivity analysis including only women with OGTTs (*N* = 3,079), no association was detected between GDM and infections (715/2,772 (25.8%) *vs.* 50/227 (22.0%) *vs.* 21/80 (26.3%); *p* = 0.452).

In multivariable logistic adjusted analysis, the use of oral or vaginal antibiotics was associated with the 2.69– and 6.99–folds, multiparity 1.44–fold and higher education level 1.35– to 1.50–folds higher risk for yeast infections (Table 3). Respective values for recurrent infection were 2.11– and 3.74–fold for oral or vaginal antibiotics, and 1.99–fold for multiparity, consequently. In subanalysis, exclusion of women without OGTTs (*N* = 886), the use of oral or vaginal antibiotics was associated with the 2.35– and 6.92–folds, multiparity 1.71–fold, and higher education level 1.68– to 1.76–folds higher risk for yeast infection. Respective values for recurrent infection were 2.20– and 3.43–fold for oral or vaginal antibiotics and 2.34–fold for multiparity, consequently. Gestational diabetes or pregestational diabetes were not associated with the yeast infections in multivariable model.

No significant associations were detected between mode of delivery, preterm birth and neonatal outcomes and yeast infections in multivariable analysis (Table 4). The mean gestational age at birth was 39.3 (SD 1.7) weeks and mean birth weight was 3,504 (SD 511) grams.

## Discussion

In this study, we did not observe associations between GDM and vulvovaginal yeast infections during pregnancy in univariate or multivariable analysis**.** The use of antibiotics (either vaginal or oral) were the most significant factors that were associated with the maternal yeast infections during pregnancy. Other related factors were maternal multiparity and higher education level. In addition, no adverse significant relation was noted between maternal yeast infections and birth outcomes. However, pregnant women with recurrent infections tended to be delivered more often by Cesarean.

In Finland, OGTT is performed on the majority of pregnant women according to the national guidelines, with GDM diagnosis seen as effective. In this study, OGTT was performed more often in women with recurrent yeast infections, which is understandable – since women with recurrent yeast infection had also higher BMIs and were thus more tested. The treatment and control of GDM as well as of DM is high-standard and free for Finnish pregnant women. As a result, blood glucose levels usually stay within safe values during pregnancy and are higher than reference values for a short period only [17]. This might explain why we found no association between GDM and vulvovaginal yeast infections in our pregnant population. Further, results were not changed even we excluded all women without OGTTs. During pregnancy, vaginal microbiota is less abundant but more stable and more *Lactobacilli*-dominant than for non-pregnant women [21–23]. These changes in vaginal microbiota may prevent yeast infections and explain rates not being significantly higher in women with GDM. We also did not observe an association between pregestational diabetes or high blood glucose mean values and increased occurrence of yeast infections, despite that such association had been reported in earlier studies in non-pregnant women [2, 7, 8]. Pregnant women with pregestational diabetes are urged to control their glucose values more strictly, than they usually do before the pregnancy, which might affect results. Possibly more severe disturbance of blood glucose metabolism is associated with the increased risk for yeast infections and pregnant women with pregestational diabetes with poor glucose levels might not participate voluntarily in cohort studies as eagerly as those with better glucose levels. Further studies must confirm these theories.

Multiparous women and those with a high number of earlier pregnancies tended to have more frequent yeast infections than nulliparous women, or women in their first pregnancies. Sasikala and associates detected a similar association between higher parity and vulvovaginal yeast infection [24], but there are no other previous studies addressing this subject. This association have been earlier related to a higher BMI in multiparous women and those with a higher number of earlier pregnancies. However, in our study obesity and multiparity associated nearly independently in multivariable analysis related to the recurrent yeast infection, so there are other explanations to this association.

There is some evidence that yeast infections during pregnancy may be associated with the development of chorioamnionitis and preterm birth, and due to prematurity and infection, these complications could lead to neurodevelopmental impairment in a newborn with low birthweight [25, 26]. Recurrent yeast infection may increase the risk of premature birth and low birthweight, even in the absence of chorioamnionitis [27]. In rare cases, vulvovaginal candidiasis has even caused maternal septicemia, [28, 29] and some studies suggest that fungal colonization could also cause septicemia in the unborn child [30]. In this study, we did not detect association between recurrent vulvovaginal yeast infections and preterm birth in multivariable adjusted analysis. Notably, women with yeast infections had used oral antibiotics more than twice as often and vaginal antibiotics nearly six times more often during pregnancy than women without yeast infections. It is possible this contributes to the lack of complications, as the risk of maternal sepsis caused by candida and premature birth is found to decrease if yeast infection was treated properly with antifungal medication during pregnancy [6, 28]. Use of oral antimicrobial antibiotics is an evident risk for development of vaginal yeast infections; yet, we did not know whether self-reported use of vaginal antibiotics included antifungal medication.

The obvious strength of this study is the large sample size. Also, the multiple number of confounders provides a comprehensive review of the subject. Some limitations must be considered, though. The majority of data was collected via electronic questionnaires, including frequency of vulvovaginal yeast infections. Since we do not know for sure whether individual yeast infections were verified by clinical examination or laboratory tests, prevalence of yeast infections may not be completely reliable. Previous studies suggest that the accuracy of self-reported yeast infections is variable [31, 32]. However, in Finland pregnant women have minimum of two free gynecological pelvic examinations during pregnancy; usually in the first and in the third trimesters. Further, women with asymptomatic yeast colonization should not be treated and tested at all, so we believe that the diagnosis of vulvovaginal yeast infections during pregnancy has been made using a combination of clinical signs and symptoms and pelvic examinations. Since OGTT was not performed for all pregnant women, it is possible that some women with possible GDM stayed undiagnosed. Yet, the prevalence of GDM (19.8%) in this study corresponds with the Finnish national prevalence in 2019 (19.1%) [33] and based on abnormal results of OGTT. The participation rate in the KuBiCo study have varied annually between 32% to 43.5% among all pregnant women who delivered at KUH. It is speculated that women in the study do not represent all pregnant women in our hospital area. However, the values of common demographic maternal characteristics, such as mean maternal age, as well as mean maternal BMI in the first trimester, corresponded well with the national Finnish demographic statistics in 2019 (31.2 years and 25.3 kg/m^2^, respectively), as with the values of 17 000 women who delivered in KUH between the years 2014–2021 (mean values 30.5 years and 25.7 kg/m^2^) [33]. Nevertheless, we had less multiparous women in this study compared to our normal parturient population (26.3%) and this may account for some selection bias in this study.

## Conclusions

We showed no association between GDM and maternal vulvovaginal yeast infections in multivariable analysis. Women with yeast infections were more often multiparous with higher education and had used more often antibiotics during pregnancy compared to others. In addition, the selected birth outcomes were favorable regardless of maternal vulvovaginal yeast infections.

## Data Availability

The dataset generated during the current study are not publicly available due to the risk of identifying patients but are available from the corresponding author on reasonable request.

## Abbreviations

BMI: Body mass index
DM: Diabetes mellitus
GDM: Gestational diabetes
ICD-10: International Statistical Classification of Diseases and Related Health Problems 10^th^ revision
KuBiCo: Kuopio Birth Cohort
KUH: Kuopio University Hospital
OGTT: Oral Glucose Tolerance Test
SD: Standard deviation
